# Silk wastes and autoclaved degumming as an alternative for a sustainable silk process

**DOI:** 10.1038/s41598-023-41762-6

**Published:** 2023-09-15

**Authors:** A. Gaviria, Natalia Jaramillo-Quiceno, Antonella Motta, Adriana Restrepo-Osorio

**Affiliations:** 1 Grupo de Investigación sobre Nuevos Materiales - GINUMA, Universidad Pontificia Bolivariana, Circular 1a 70-01, 050031 Medellín, Colombia; 2 Grupo de Investigaciones Agroindustriales - GRAIN, Universidad Pontificia Bolivariana, Circular 1a 70-01, 050031 Medellín, Colombia; 3 BIOtech Research Centre and European Institute of Excellence On Tissue Engineering and Regenerative Medicine, Department of Industrial Engineering, University of Trento, Via Delle Regole 101, 38123 Trento, Italy; 4 Facultad de Ingeniería Química. Escuela de Ingenierías, Universidad Pontificia Bolivariana, Medellin, Colombia

**Keywords:** Biomaterials, Environmental impact

## Abstract

Silk degumming is considered the first point in the preparation of silk-based materials since this process could modify the silk fiber and the properties of its related products. This study evaluated the differences in morphology, secondary structure, amino acid content, thermal stability, and mechanical properties of two types of raw materials, defective cocoons (DC) and silk fibrous waste (SW), degummed by chemical (C) and autoclaving (A) methods. Subsequently, silk fibroin films were prepared by dissolving each type of degummed fibers, and thermal and structural films properties were determined. The findings demonstrated that autoclaving is an efficient alternative to remove silk sericin, as the resulting fibers presented improved structural, thermal, and mechanical properties compared to those obtained by the chemical method. For films preparation, autoclave resulted in a good option, but dissolution parameters need to be adjusted for defective cocoons. Furthermore, similarities between the physicochemical properties of fibers and films from both fibrous wastes suggest that SW is a promising raw material for producing fibrous resources and regenerated silk fibroin materials. Overall, these findings suggest new recycling methods for fibrous waste and by-products generated in the silk textile production process.

## Introduction

Silk is a natural protein consisting of two proteins, specifically, silk fibroin (SF) fibers surrounded by a sericin layer. Silk sericin (SS) must be removed by degumming to improve textile spinning and dyeing processes. Several methods have been studied for degumming based on aqueous solutions containing acids, alkalis, or enzymes to hydrolyze the SS. Chemical degumming with sodium carbonate (Na_2_CO_3_) is the most widely used method because of its high percentage of removed sericin^1^; however, this process is energy-consuming, and the SS contained in the degumming wastewater is difficult to recover. Alternative degummed processes, such as autoclave, microwave or ultrasonication process, are considered more environmentally friendly as they reduce chemical waste and allow for silk sericin recovery compared to traditional chemical processes^2–4^. According to some estimates, the energy demand in the silk textile process is approximately 1800 MJ per kilogram of fiber, which is significantly higher than that required for other fibers, such as nylon or cotton yarn. Only the cocoon cooking process consumes 51% of the energy required for raw silk production. Besides, it was stated that one of the most significant emissions in the sericulture process is due to the discharge of chemicals and SS in drainage systems and groundwater during chemical degumming^5^. In fact, in the processing of 400 thousand tons of dry cocoons in the silk textile industry, some 50 thousand tons of sericin are generated and discarded^6^.

Additionally, heterogeneous fibrous waste is obtained during the silk textile process, and because of its nature, it cannot be used in conventional textile production. These fibrous wastes could be classified as defective cocoons (DC) and silk fibrous waste (SW). DC includes double cocoons, non-reeling, stained, and deformed cocoons, and silk fibrous waste (SW), which is a heterogeneous residue composed of proportions of DC, short threads (outer floss), the innermost layer of the cocoon (pelade layer), and some portions of yarns. Both types of silk fibrous waste are of interest in research due to their fibroin content and possible specific applications such as medical devices, cosmetics, pharmaceuticals, optics, foods, biosensors, and electronics^7^. These residues represent approximately 35% of the total silk produced worldwide^8^, and approximately 11 million tons of silk waste are produced globally every year^6^. Specifically, for the Colombian case, in the Corporation for the Development of Cauca Sericulture (CORSEDA), silk waste represented between 20 and 30% of total production.

In previous studies, the effects of degumming on the properties of silk fibers have been reported^1,6,7^. However, only a few studies have compared the effects of autoclaving and sodium carbonate (Na_2_CO_3_) degumming on the properties of DC and SW. Furthermore, owing to rapid population growth, the demand for resources has increased, including textile garments such as silk. For this reason, there is a need to study the recycling of silk waste and evaluate alternatives for more environmentally friendly degumming processes. This information is essential since these wastes have a high potential for obtaining fibrous materials and products derived from regenerated silk fibroin, such as films, foams, and electrospun materials^8,9^. Therefore, this study proposes an evaluation of the influence of two degumming methods, autoclave (A) and chemical (C), on the physicochemical properties of two different types of raw materials to obtain silk fibers and silk fibroin films: defective cocoon (DC) and silk fibrous waste (SW) from the *Bombyx mori* silkworm.

## Results

### Fibers characterization

The percentage of mass loss after each degumming process was determined as a quantitative measure of silk sericin removal. The average percentages and its standard deviations were by the chemical method SW-C: 4.12 ± 0.2% and DC-C: 25.62 ± 0.55%, by autoclave were SW-A:2.82 ± 1.74% and DC-A:24.71 ± 0.07%. The results showed a significant statistical difference with a p-value < 0.001 for the degumming percentage when comparing the raw materials (SW and DC), but there was no significant statistical difference for the comparison related to the degumming process.

**Figure 1:**
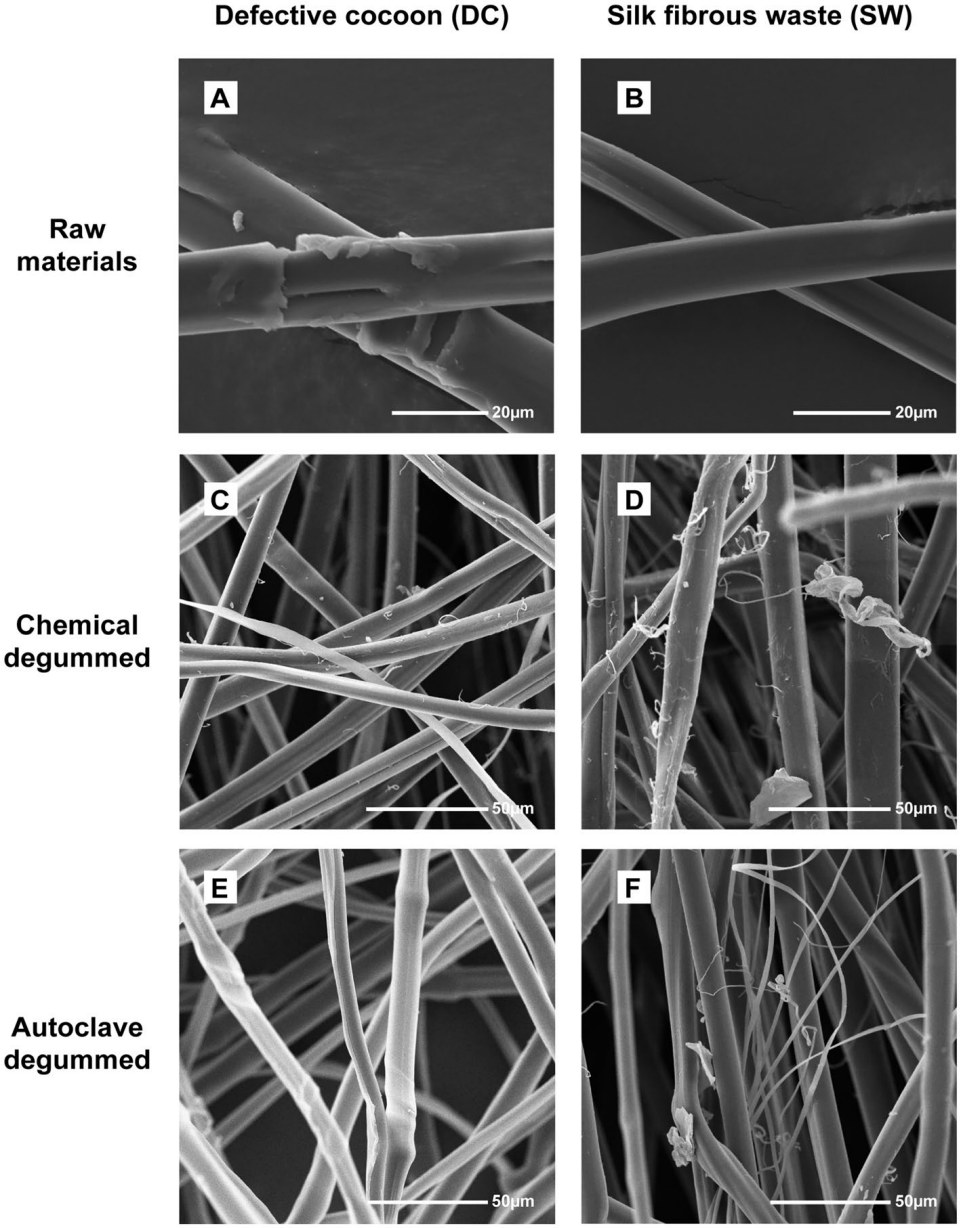
SEM images for (**A**) SW, (**B**) DC, (**C**) SW-A, (**D**) SW-C, (**E**) DC-A, and (**F**) DC-C.

Morphological changes were observed using scanning electron microscopy (SEM) images at 2000×, 1000× and 500 magnification for both DC and SW before and after degumming using both methods (Fig. 1 and S1). DC fibers (Fig. 1-A) exhibit sericin coating, while SW (Fig. 1-B) presents a lower amount. When comparing fibers degummed by sodium carbonate and autoclave, SW-C (Fig. 1-D and S1-B) shows superficial fibrillation and a comparatively damaged structure compared to DC-C (Fig. 1-C and S1-A), and a similar behavior was observed for SW-A (Fig. 1-F and S1-D) and DC-A (Fig. 1-E and S1-C).

**Figure 2:**
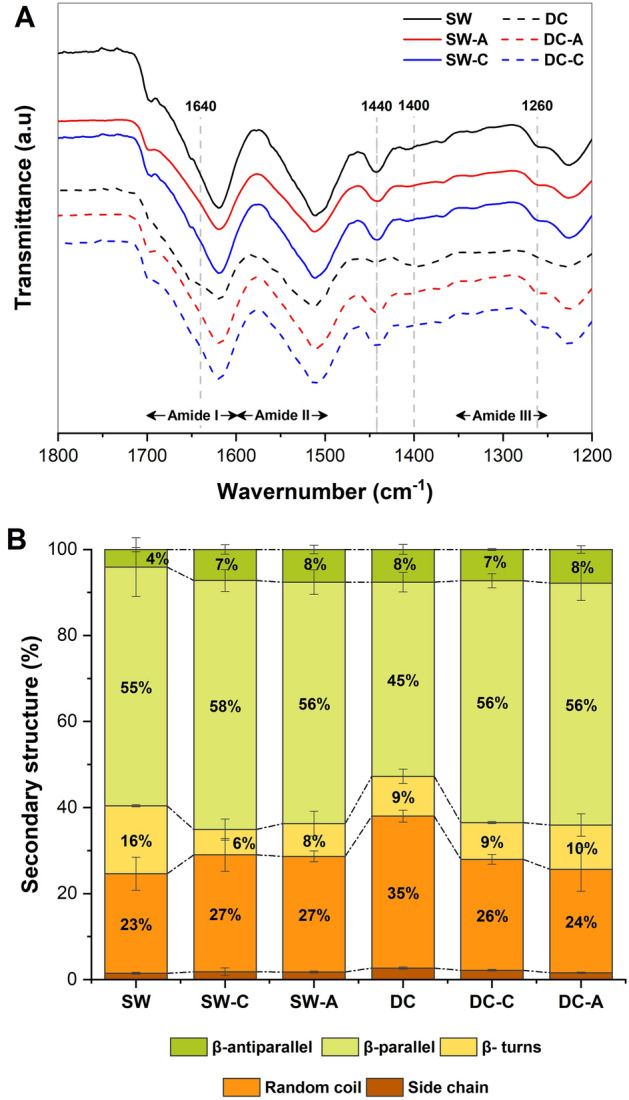
(**A**) FTIR spectra, and (**B**) secondary structure (%) of SW, DC, SW-A, SW-C, DC-A, and DC-C.

Fourier transform infrared spectroscopy (FTIR) is a technique that allows the study of the secondary structure of proteins based on the principle that hydrogen bonds are associated with elements of the secondary structure and have characteristic IR absorption frequencies^13^. FTIR spectra obtained in Fig. 2-A showed the characteristic bands for silk fibroin in all samples: ~ 1643 cm^−1^ (amide I), ~ 1518 cm^−1^ (amide II), and ~ 1234 cm^1^ (amide III). Only the DC sample showed bands at 1400 cm^−1^ and 1640 cm^−1^, associated with the presence of sericin^16^ and random coil conformations^14^, respectively. In this sample, the absence of bands at 1260 cm^−1^ and 1440 cm^−1^ suggested a lower presence of antiparallel β sheet structures^12,13^.

Individual band areas in the spectra were determined to compare and calculate the relative contributions of each secondary structure in all samples. As seen in Fig. 2-B, no statistically significant difference was found when comparing the degummed samples obtained by both methods. However, with a p-value < 0.05, a statistically significant difference was found when comparing both raw materials (SW and DC), in random coil (16.46% and 33.63%) and β-turn structures (8.81% and 24.20%) specifically.

Amino acid analysis was used to determine its content in the samples and relate it to properties such as moisture absorption and biodegradation that occur mainly in the amorphous regions of silk. This is related to the presence of polypeptide molecules with a fraction of bulky side groups or, on the contrary, with small and simple side groups that allow the arrangement in an orderly and crystalline manner, which will influence the desirable mechanical, physical, and chemical properties of silk fibers^17^. The amino acid content of all samples is shown in Table 1.

**Table 1:** Amino acid content of SW, DC, SW-A, SW-C, DC-A, and DC-C.

Amino acid residues	Samples					
	DC	SW	DC-A	DC-C	SW-A	SW-C
Nonpolar amino acids (hydrophobic)						
Glycine*	**47.26**	**53.48**	**54.11**	**54.49**	**53.00**	**56.26**
Alanine*	**26.74**	**32.79**	**28.24**	**29.87**	**27.06**	**30.07**
Valine	2.61	2.43	2.09	2.26	2.06	2.05
Isoleucine	1.12	1.23	1.03	1.13	0.96	0.97
Leucine	0.75	0.66	0.56	0.61	0.56	0.53
Phenylalanine	0.68	0.82	0.63	0.68	0.66	0.62
Proline*	0.54	0.48	0.42	0.47	0.42	0.41
Polar amino acids (hydrophilic)						
Serine*	**5.89**	**2.22**	**5.68**	**3.74**	**5.93**	**3.93**
Tyrosine	4.48	4.63	4.26	4.67	4.46	4.31
Threonine*	1.51	0.18	0.86	0.49	0.60	0.60
Polar-charged side chains (hydrophilic)						
Acidic—negative charged						
Aspartic acid	**5.80**	**0.00**	**1.31**	**0.77**	**2.61**	**0.00**
Glutamic acid	0.83	0.00	0.44	0.00	0.96	0.00
Basic—positive charged						
Arginine	0.90	0.45	0.00	0.48	0.39	0.00
Lysine	0.87	0.33	0.37	0.35	0.32	0.26
Histidine*	0.00	0.31	0.00	0.00	0.00	0.00
Hydrophobic	79.7	91.89	87.08	89.51	84.72	90.91
Hydrophilic	20.28	8.12	12.92	10.5	15.27	9.1
Bulky	18.04	10.55	10.69	10.95	12.98	8.74
Non-bulky (*)	81.94	89.46	89.31	89.06	87.01	91.27

* Refers to non-bulky amino acid residues. Numbers in bold refer to the main changes in aminoacids.

**Figure 3:**
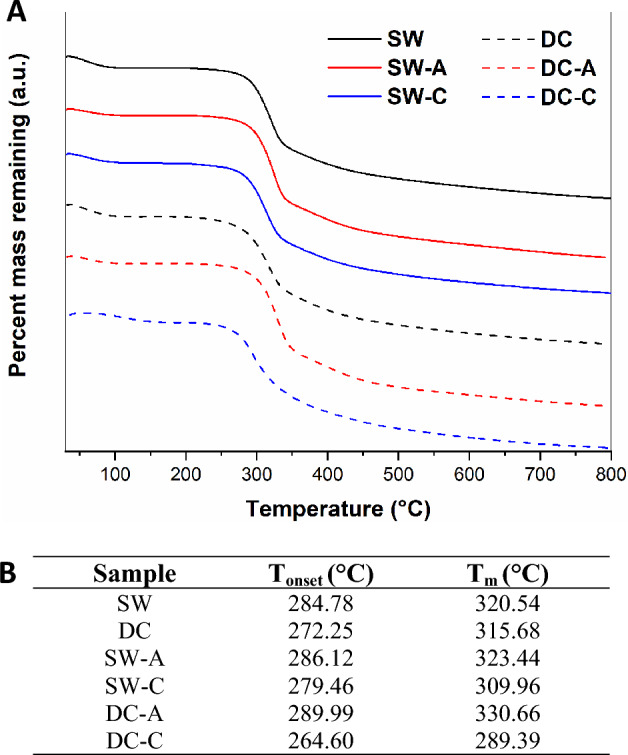
(**A**) TGA thermograms, and (**B**) TGA results for SW, DC, SW-A, SW-C, DC-A, and DC-C.

Thermogravimetric analysis (TGA) measures the change in the mass of a sample as the temperature increases at a constant rate. When studying silk proteins, this technique has been useful for quantitatively evaluating the changes in water content with increasing temperature, in addition to the degradation initiation temperature (T_onset_) and maximum degradation temperature (T_m_)^13^. In Fig. 3-A, two thermal events were observed: the first one at approximately 100 °C was attributed to the loss of water in the sample, and the second at approximately 300 °C was due to the degradation of SF.

When comparing the raw materials, a higher T_onset_ and T_m_ for SW were observed compared to DC; additionally, the DC-A and SW-A samples presented higher T_onset_ and T_m_ compared to the chemically degummed samples (Fig. 3-B). This could be due to lower conformational mobility for the prevalence of crystalline structures^13^.

Modulated differential scanning calorimetry (MDSC) is a thermal analysis technique in which the normal temperature scan used in DSC is typically overlaid with low-frequency sinusoidal perturbation. The advantages of MDSC include improved resolution and sensitivity, as well as the ability to separate overlapping phenomena. It can be used to study reversible and non-reversible heat flow, clarifying the effects of non-reversible events, such as the dewatering of silk samples^13^.

**Figure 4:**
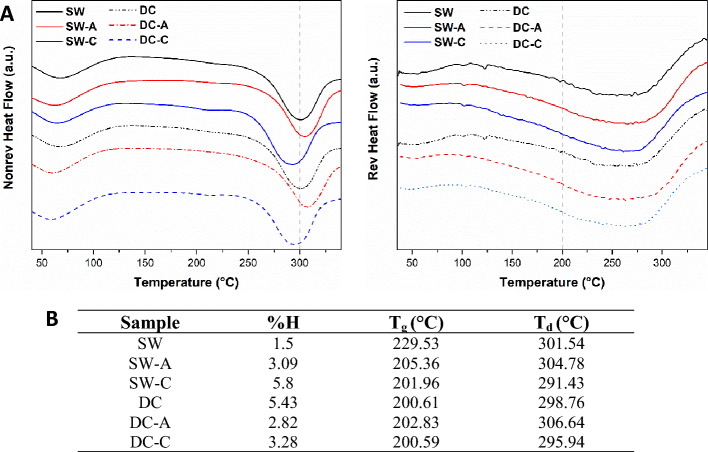
(**A**) MDSC thermograms, and (**B**) MDSC results for SW, DC, SW-A, SW-C, DC-A, and DC-C.

The MDSC thermograms in Fig. 4-A show the reversible process of the glass transition temperature (T_g_), humidity percentage (%H), and decomposition temperature (T_d_) for the non-reversible process. Percentage values, T_g,_ and T_d_ for all samples were compared with the previous trend (Fig. 4-B). Analyzing the effects of degumming, the percentage values of humidity exhibited the following trend from lowest to highest: DC-A < SW-A < DC-C2 < SW-C2. In addition, the trends for T_d_ and T_g_ responded to inverse behavior, where SW-C2 < DC-C2 < SW-A < DC-A.

**Figure 5:**
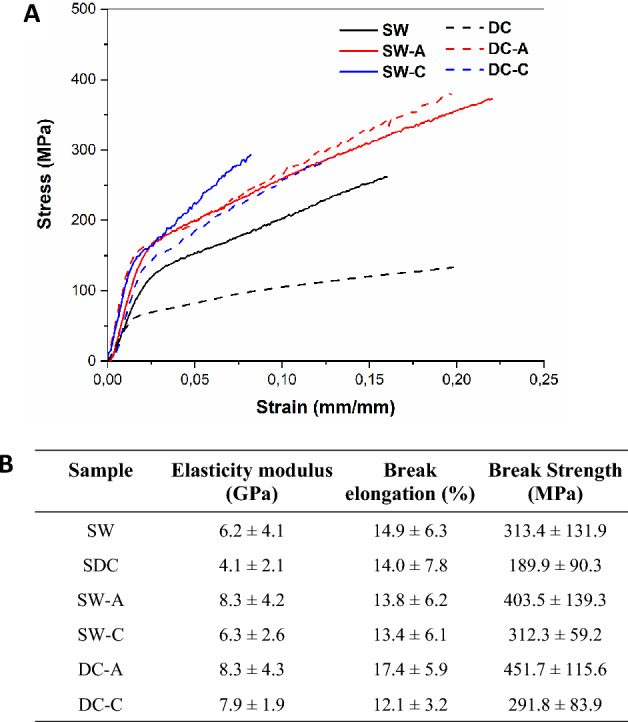
(**A**) Stress–Strain tensile curves, and (**B**) mechanical behavior of SW, DC, SW-A, SW-C, DC-A, and DC-C.

It is important to highlight silk fiber as one of the strongest natural fibers, with outstanding mechanical properties, high strength, stiffness, and toughness under both tension and compression loading. Because degumming imposes a markedly unnatural environment on silk, it is important to consider the possibility of changes in the fibroin structure and mechanical properties^15,16^. Figure 5-A shows representative stress–strain curves for the SW, DC, SW-A, SW-C, DC-A, and DC-C samples. All sample types exhibited ductile behavior, where silk fibers could be elongated by a high percentage before breaking. However, the average values for break strength, elasticity modulus, and break elongation are larger for samples SW, SW-A, and DC-A than for DC, SW-C, and DC-C, respectively (Fig. 5-B).

In addition, box plots in Supplementary Figure S2-A and C for elasticity modulus and break strength, respectably, exhibited higher averages but greater deviations in cocoon fibers compared with waste fibers. For break elongation in S2-B no significant differences are observed. In addition, the marginal means plot for break strength in S2-D shows that the ANOVA values comparing raw materials present a significant difference (p-value < 0.01). The marginal means plot for break strength in S2-H follows the same ANOVA trend, also showing a significant difference when comparing the degumming type as well as higher averages when comparing box plots in S2-E, F, and G for elasticity modulus, break elongation and break strength of samples degummed by autoclave.

### Films characterization

Films of regenerated silk fibroin were obtained by casting from the degummed fibers of silk waste and silk cocoons through chemical and autoclave processes, F-SW-A, F-DC-C, F-SW-A, and F-DC-C, respectively.

**Figure 6:**
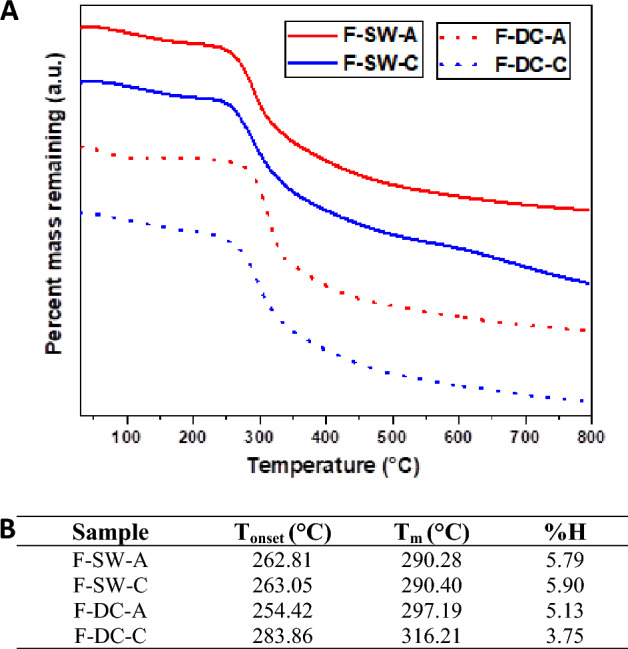
(**A**) TGA thermograms, and (**B**) TGA results for F-SW-C, F-SW-A, F-DC-C, and DC-A.

In the TGA thermograms (Fig. 6-A), two thermal events were observed; the first one around 100 °C was attributed to the loss of water in the sample, and the second around 300 °C was due to the degradation of SF. TGA results (Fig. 6-B) doesn’t significant difference presented for films obtained from chemically and autoclaved degummed silk waste, in films obtained from cocoon silk, sample F-DC-C showed a significantly higher T_onset_ and T_m_ than the other films.

**Figure 7:**
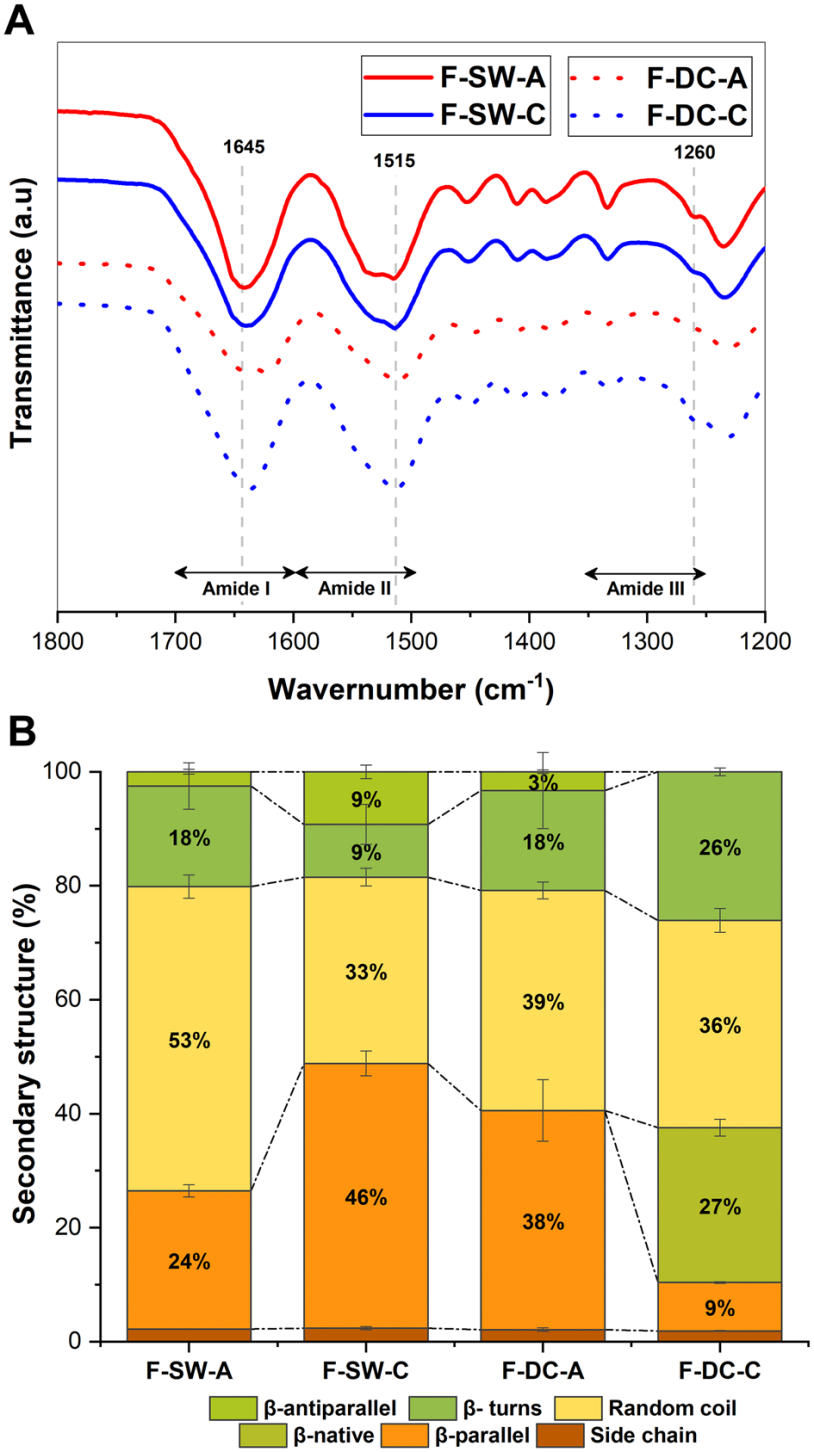
(**A**) FTIR spectra, and (**B**) secondary structure (%) for F-SW-C, F-SW-A, F-DC-C, and DC-A.

FTIR spectra for films obtained in Fig. 7-A showed the characteristic bands for silk fibroin in all samples: ~ 1643 cm^−1^ (amide I), ~ 1518 cm^−1^ (amide II), and ~ 1234 cm^1^ (amide III). Additionally, a band at 1645 cm^−1^ for random coil structures and at 1260 cm^−1^ and 1515 cm^−1^ for antiparallel β-sheet structures and parallel β-sheet conformations, respectively, suggested a higher crystallinity for cocoon samples compared to the films obtained from waste^12,17,18^. Indeed, the analysis in the amide I region (Fig. 7-B) showed the presence of native β-sheet crystalline structures in sample F-DC-C, confirming the results obtained in the TGA for this sample and highlighting its high crystallinity.

## Discussion

The chemical degumming process usually needs to be repeated several times to completely remove sericin, resulting in higher energy consumption, increased cost in chemical products, and an additional purification process for recovering the SS from the generated wastewater^17–19^. Cocoon and silk waste fibers were degummed by chemical and autoclave methods, and it was concluded that the degumming method and raw material type affect the mechanical properties of SF fibers, and these properties can be improved by controlling the degumming method^6,20^. The results showed that both types of raw materials could be used to obtain fibrous resources and regenerated fibroin, an area of great interest for the development of different silk-based materials, in addition to the use of a residue that is currently not fully exploited in the silk industry, where recycling and utilization of SW should evolve in the direction of high performance, low cost, and cleaner production.

Regarding degumming percentages, autoclave and chemical processes presented comparable sericin removal; however, there was a statistical difference between the evaluated raw materials (SW and DC). Similar results have also been reported; about no differences between the percentages of sericin removed in both degummed methods (approximately 22%), and sericin can be hydrolyzed and becomes highly soluble in water, resulting in its separation from SF^22^. Differences between raw materials could be attributed to the nature of wastes because SW involves previous physiochemical treatments that would result in a lower presence of sericin^26,27^, as can be seen in the SEM images, where for DC, two SF fibers coated by a sericin layer can be observed compared to SW with individual SF fibers. This finding related to SW could be considered an advantage over defective cocoons because degumming processes would be less aggressive, with lower energy requirements and less use of reagents that will later be released into wastewater. Both degumming methods remove the existing sericin; however, SEM images show increased fibrillar damage in chemically degummed SW and DC. Previous studies have reported similar results when comparing these degumming methods, indicating that chemical degumming may cause hydrolytic degradation of proteins^21,22^.

The FTIR results confirmed the differences attributable to the presence of SS, with the DC spectra presenting well defined characteristic bands of SS, which are related to a higher percentage of amorphous structures. Raw material comparisons showed differences in β-turn and side chains, corresponding to crystalline and amorphous secondary structures, respectively. This may be due to the DC having not undergone any prior SS removal process, shows an amorphous random form^21,22^. Although there were no significant differences between the chemical and autoclave degummed samples, a decrease in the β-turn structure was observed. The β-turn structure is a short hydrophilic chain that connects β-sheet structures, and its decrease suggests that the degumming process with Na_2_CO_3_ causes partial loss of the β-turn structure. This indicates that this degumming method causes more damage to the silk structure and reduces the content of crystalline structures^31^, in agreement with the SEM results.

The amino acid content analysis indicated to the DC and autoclave degummed samples (DC-A and SW-A) contained a higher percentage of serine (Ser) and aspartic acid (Asp) than SW, DC-C, and SW-C, which present a higher percentage of glycine (Gly) and alanine (Ala). Ser and Asp are related with a higher presence of SS meanwhile Gly and Ala are characteristic aminoacids associated to SF^23,26^. This corresponds with the observed trend in SEM and FTIR results, as it confirms a higher SS content in the DC and autoclave degummed samples, while SW and chemically degummed samples have a higher ratio of silk fibroin (SF).

Additionally, the thermal analysis suggests that SW has major thermal stability compared to DC raw material; this could be attributed to a lower presence of SS in the SW sample compared to the DC sample due to the previously mentioned processes and a lower crystallinity, as evidenced by SEM, FTIR, and amino acid analysis. In the degummed comparison, the autoclave samples exhibited greater T_onset_, T_m_, T_g_, T_d_, and lower humidity percentage than the chemically degummed samples. These findings corroborate the SEM images, where fibers degummed by autoclave show less surface wear, and the results found in FTIR, where a higher content of crystalline structures was observed in the autoclave degummed samples compared to the chemically degummed samples. The aforementioned is related to a greater thermal stability, lower degradation rate, lower moisture absorption, and also with the improve mechanical behavior and the biodegradation rate^14,25^.

Mechanical testing showed that the elasticity modulus and the break strength of the SW and autoclave degummed samples (SW-A and DC-A) were better than those of the DC and chemically degummed samples (SW-C and DC-C). This behavior is related to the higher crystallinity observed in the autoclave samples which suggests that high temperatures promote new crystalline structures^33^. However, only break strength presented a statistical difference for both compared groups, material and degummed types, indicating as previous report, that the reorganization of the secondary structures related to the evaluated degummed methods did not significantly modify the mechanism by which the fibers were stretched; however, it did have a statistically significant effect on their tensile strength^33^.

The results indicated that SW has higher thermal and structural stability, as well as better mechanical behavior than DC, which could be due to the lower content of SS present in the SW sample. Nevertheless, this last type of raw material presents a heterogeneous and pretreated nature of the residue. Additionally, the finding results show that autoclaving causes less fibers damage, maintains its thermal stability, and promote the formation of crystalline structures, which is improves its mechanical properties compared to the results obtained for fibers degummed by chemical method. This is consistent with results reported by other researchers that found that SS can be removed by autoclaving without the need for chemicals, obtaining equal or improved degumming percentages and fibers properties^34^.

In the thermal analysis of films obtained from degummed fibers, higher thermal stability was observed for the F-DC-C. This behavior is similar for sample F-DC-A; but the T_onset_ of this sample did not follow the expected trend. This could be due to heterogeneity in the removal of sericin, as discussed previously by other authors^35^. At the end of degumming, some portions of the fiber may retain SS remnants, whereas other parts, which initially lacked this protective SS layer, may suffer greater structural damage^36^. In fact, this heterogeneous trend was related with the higher dispersion in mechanical results for fibers degummed by autoclave compared to chemically degummed fibers for both raw materials.

Furthermore, as reported in the methodology section, sample F-DC-A could not be dissolved using standardized protocols, and was necessary to reduce the SF:LiBr bath ratio. This was related to a higher percentage of SS present in this sample, as previously mentioned in the discussion of the results for amino acid content and SEM images of the fibers, as shown in Supplementary Figure S1, by increasing the observation scale.

The FTIR analysis of the secondary structures in SF films did not show statistical differences when comparing the type of degumming. However, when comparing the raw materials, differences in crystallinity between the regenerated SF films obtained from the DC and SW were confirmed (p-value < 0.001). These results align with the fiber analysis and demonstrate the suitability of both degumming methods for fiber degumming and SF production. However, to maintain an environmentally sustainable approach that enables the recovery of sericin in DC degummed by an autoclave process, it is recommended to assess new parameters process.

## Conclusion

The use of silk waste to obtain fibrous materials and regenerated fibroin has great potential for application in the research area because of the growing interest in the circular economy, including the reuse of materials and the development of new research in the biomedical area for SF. The results of this study indicate that the physicochemical properties of silk can be modulated by the degumming method and by the source of the raw material used. Fibers and films obtained from silk wastes showed performance comparable to that of cocoon fibers in terms of structural, thermal, and mechanical properties in both types of fibers, indicating that both types of raw material could be used for fibrous material processing and production of regenerated fibroin.

Silk waste stands out as an effective alternative owing to environmental constraints and the high production of textile waste caused by high population growth. Additionally, the effects of chemical degumming were more abrasive on cocoons and waste silk fibers than on degumming by autoclaving. This degumming method presents an option to eliminate textile waste that leads to the contamination of water, soil, and crops due to the salts present in chemical degumming. The fibers degummed by autoclaving showed less surface wear, a higher percentage of crystallinity, greater thermal stability, and better mechanical response; meanwhiles the SF obtained from these fibers exhibited comparable properties for both degumming methods.

The implications of this study include the strengthening of new degumming tendencies that are environmentally sustainable, such as autoclave degumming, allowing the reduction of chemical waste and the high energy requirements of chemical degumming. However, the autoclave process requires high-investment equipment and specialized management; therefore, its application in the textile process could be included in the long term with several initial implementation processes. Additionally, for SF obtained from defective cocoons degummed by autoclaving, it is recommended to assess new parameters process. In this sense, further studies on production could focus on the implementation of autoclave degumming in the traditional silk textile chain and research on the comparison of regenerated fibroin obtained from silk waste and its comparison with cocoon-based fibroin with a perspective in biomedical applications.

## Materials and methods

### Degummed method

DC and SW donated by the Corporation for Development of Sericulture in Cauca (CORSEDA) were degummed using two conventional methods: chemical (C) and autoclave (A). The chemical method used was alkaline degumming, and silk was boiled in 0.5% (w/v) Na_2_CO_3_ solution with a liquor ratio of 1:100 for 30 min and was repeated twice. Autoclave degumming was performed at a liquor ratio of 1:50 for 50 min at 121 °C. For both methods, degummed silks were washed with distilled water and dried at 60 °C. The degree of degumming was calculated in terms of weight loss after different degumming methods using the following formula:

1 $$\% \mathrm{weight\, loss}=\left(\frac{{w}_{f} - {w}_{i}}{{w}_{f}}\right)*100$$

where: $${w}_{i=}weight\, before\, degumming$$, $${w}_{f=}weight\, before\, degumming$$.

### Silk fibroin extraction and film formation

Degummed silk fiber was dissolved in a 9.3 M Lithium Bromide (LiBr) solution in a bath ratio of 20:100 SF:LiBr for 4 h at 60 °C, followed by dialysis and filtration. A bath ratio of 10:100 was used for the F-DC-A sample. The obtained solution was casting dried for 24 h at 35 °C to obtain the SF films.

### Scanning electron microscopy (SEM)

The morphology of the degummed silk fiber was observed by Scanning Electron Microscopy (SEM, JEOL JSM-6490 LV) after gold coating at 15 kV. Fiber diameters were measured in triplicate in three different parts. The means and standard deviations were reported.

### Fourier transform infrared spectroscopy (ATR-FTIR)

The secondary structure of the silk fibers was identified using attenuated total reflectance Fourier transform infrared spectroscopy (ATR-FTIR). For each measurement, 32 scans were collected at a resolution of 1 cm^−1^, in the range 650–4000 cm^−1^. Fourier self-deconvolution (FSD) of the Amide I region was performed using OriginPro® software. The deconvoluted spectra were fitted with seven Gaussian curves to compare the effect of the different degumming processes on the secondary structure of the fibers according to the methodology presented by Bucciarelli et al.^37^. Triplicates were analyzed to determine the percentages of crystalline structures in each sample, and one-way analysis of variance (ANOVA) was used to evaluate the differences between samples.

### Amino-acid composition

Amino acid analysis was carried out using the Waters AccQFluorTM Reagent Kit and Waters AccQ.TagTM amino acid analysis method. The samples were hydrolyzed in 6 N HCl at 114 °C for 24 h. The hydrolysate was diluted with 20 mM HCL and mixed with Waters AccQ-Flour Reagent. The amino acid content of one sample for each experiment was determined by reversed-phase high-performance liquid chromatography (RP-HPLC) using a Jasco UV-1570 detector (Jasco, Bouguenais, France) at 254 nm and 37.0 ± 0.5 °C^38^.

### Thermogravimetric analysis (TGA)

Thermogravimetric analysis (*TGA; SDTA 851*, *Mettler Toledo)* was used to measure the weight changes of the silk fibroin samples with increasing temperature*.* The test conditions included 10 mg of the sample in a temperature range of 30–900 °C, a heating rate of 10 °C/min, and an inert nitrogen atmosphere. For the thermal tests, one curve was analyzed for each main sample.

### Modulated differential scanning calorimetry (MDSC)

Modulated Differential Scanning Calorimetry (MDSC) tests were performed using a Q2000 DSC instrument (TA Instruments). In an aluminum crucible, 3 mg and 4 mg of the sample were deposited and purged with nitrogen. Tests were performed between 30 and 340 °C at a heating rate of 3 °C/min with a modulation amplitude of 0.62 °C.

### Mechanical properties

To evaluate the mechanical properties of the SF fibers, stress–strain curves were obtained using a universal testing machine (Instron 5582), according to ASTM D3822. Twelve fibers with an initial length of 40 mm were tested for each treatment. The fibers were subjected to a loading procedure using a load cell of 50 N and an extension rate of 24 mm/min. An estimate of each specimen strain was determined from the displacement of the loading system crosshead (grip-to-grip displacement). Stress at break (MPa), modulus of elasticity (GPa), and elongation at break (%) were determined. Analysis and interaction graphs were generated using the Jamovi® software.

### Statistical analysis

Data are expressed as the mean values and standard deviations. The Shapiro test was used to analyze the statistical distribution. One-way ANOVA evaluated the level of statistical significance through Welch or Fisher’s t-test in Jamovi ® software whit a p-value < 0.05 taken for statistical significance.

### Supplementary Information

Supplementary Information.

## Data Availability

The datasets generated and/or analyzed during the current study are not publicly available, as these results are associated with an ongoing project with a previously established intellectual property regulation but are available from the corresponding author upon reasonable request.
