# Supplementary material for: Silk wastes and autoclaved degumming as an alternative for a sustainable silk process
Source: Sci Rep. 2023 Sep 15;13:15296. doi: 10.1038/s41598-023-41762-6 (PMC10504296; doi:10.1038/s41598-023-41762-6)
Supplement: Supplementary file 1 — Supplementary Information. [file 41598_2023_41762_MOESM1_ESM.docx]

**Silk wastes and autoclaved degumming as an alternative for a sustainable silk process**

**SUPPLEMENTARY INFORMATION**


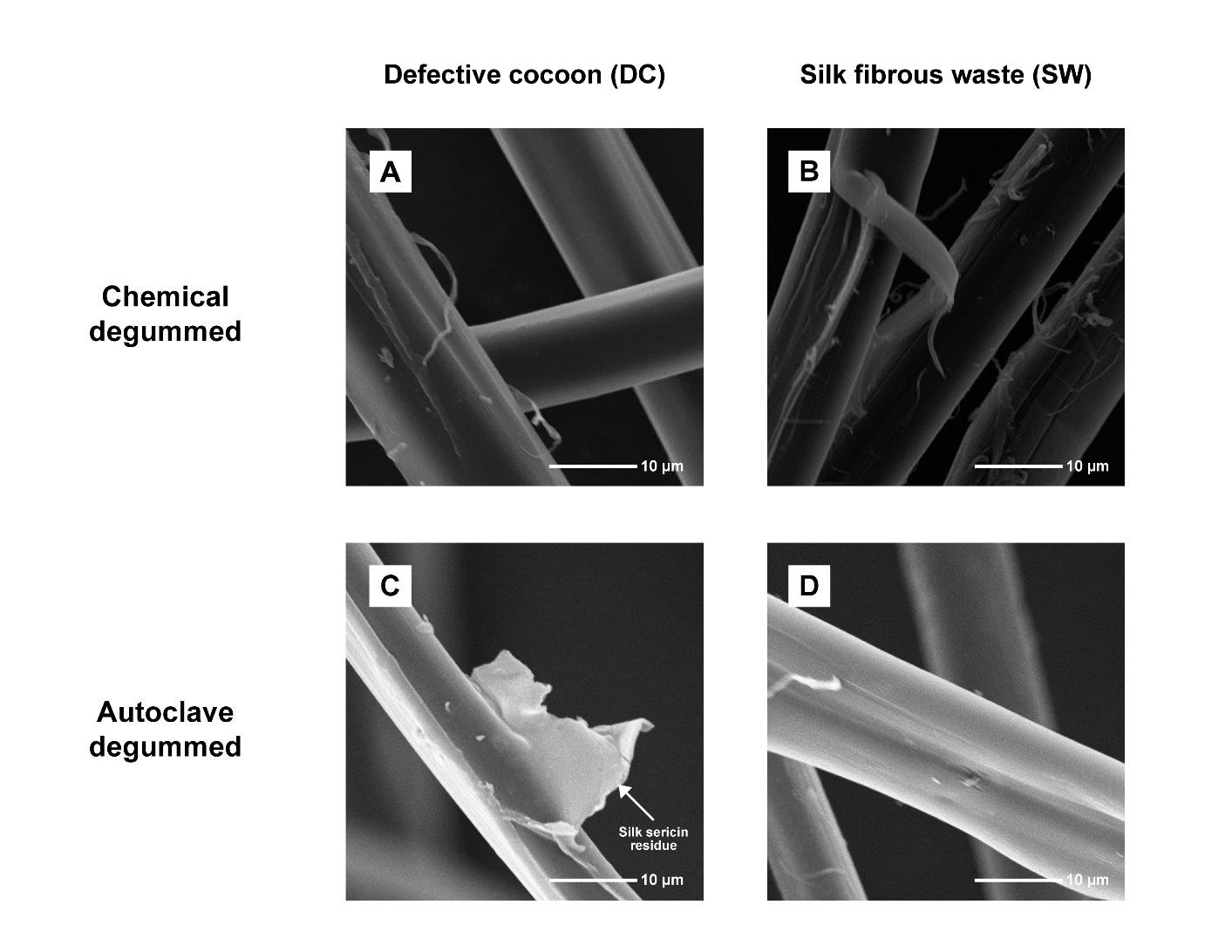


**Figure S1**. SEM images at 2000X for A: SW, B: DC, C: SW-A, D: SW-C, E: DC-A, and F: DC-C*.*


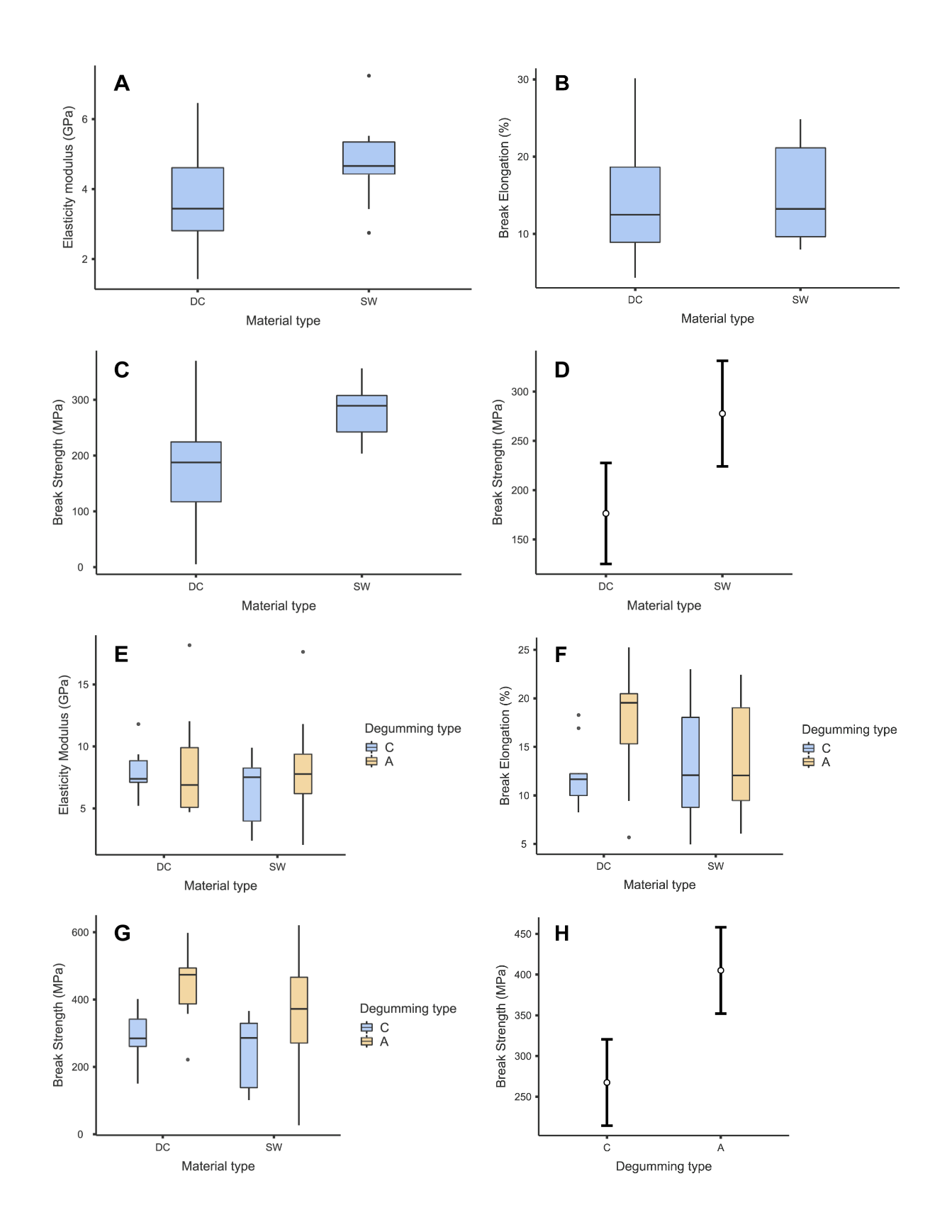
 **Figure S2**: Box plot diagrams for A: elasticity modulus, B: break elongation, C: break strength, and D: marginal means plot for break strength of SW and DC samples. Box plot diagrams for E: elasticity modulus, F: break elongation, G: break strength, and H: marginal means plot for break strength of SW-A, SW-C DC-A, and DC-C samples.
